# On-chip manipulation of trion drift in suspended WS_2_ monolayer at room temperature

**DOI:** 10.1515/nanoph-2024-0739

**Published:** 2025-03-21

**Authors:** Woo Hun Choi, Seong Won Lee, Su-Hyun Gong

**Affiliations:** Department of Physics, 34973 Korea University, Seoul, 02841, South Korea

**Keywords:** trion drift, optoelectronics, transition metal dichalcogenide, tungsten disulfide

## Abstract

Excitons, which are bound states of electrons and holes, in transition metal dichalcogenides (TMDCs) have been studied as an information carrier for realizing new types of optoelectronic devices. However, the charge neutrality of excitons inhibits the electric control of their motion, as seen in conventional electronic devices, except when utilizing a heterostructure. Here, we investigated the drift motion of trions, quasiparticles composed of an exciton bound to an excess charge, at room temperature in a suspended WS_2_ monolayer by applying a gate-tunable electric field. Using a simple bottom-gate device, we can tune the electric field intensity and exciton-to-trion conversion ratio by increasing the charge density in the monolayer. Consequently, we experimentally observed that locally excited trions drift toward the center of the suspended monolayer. To understand the underlying mechanisms, we numerically simulated the trion drift using the drift-diffusion equation, accounting for the contributions from both the electric field and strain. The results confirmed that the electric field plays the dominant role in the drift phenomena. Our work offers a useful platform for realizing trion-based optoelectronic devices that are capable of operating even at room temperature.

## 1 Introduction

Following the discovery of graphene, there has been a significant increase in interest in two-dimensional (2D) materials, especially transition metal dichalcogenides (TMDCs). Notably the discovery that MoS_2_ [1], [2] and other TMDCs exhibit a direct bandgap when reduced to monolayer thickness has sparked considerable attention [3], [4], [5]. This has been accompanied by a growing body of research focused on the diffusion, drift and transport of excitons, in these materials [6], [7], [8], [9], further highlighting their potential for optoelectronic applications [10], [11]. Since excitons are charge-neutral particles, their motion cannot be directly controlled by an external electric field, unlike charged carriers such as electrons or holes. To control their motion, an effective force must be generated by a local energy gradient [7], [8], [9], [12], [13]. However, it has been reported that exciton drift is an inefficient process, even when the maximal strain-induced potential gradient is applied [14]. To address these challenges, interlayer excitons (IXs) in Van der Waals heterostructures with type-II band alignment are employed [15], [16], [17], [18], [19], [20]. Their long lifetime and the spatial separation of charges make IXs a promising platform for realizing electrically controllable excitonic devices.

Compared to neutral excitons, trions (electron-hole pairs with an additional charge) [21], [22] can respond to an external electric field due to their excess charge, making them more easily manipulated by such fields. This characteristic offers an advantage in applications that require electrical control, opening up the possibility for the development of trion-based devices, thus advancing beyond traditional control methods. Early attempts to control the motion of trions using an electric field were demonstrated in 2001, utilizing a GaAs quantum-well structure with a lateral gate voltage, well before TMDCs gained significant attention [23], [24]. Nevertheless, studies on trion transport in 2D TMDCs have only recently begun to emerge [6], [25], [26], alongside growing efforts to improve the exciton-to-trion conversion rate [27], [28], [29], [30]. In the case of 2D TMD materials, the drift motion of trions was demonstrated under the influence of a laterally applied in-plane electric field, with the trions excited by optical pumping in monolayer WS_2_ [25]. However, optically pumped trions without electrostatic doping are bound to ionized donors, hence the maximum propagation distance is limited by the electric potential generated by its donors. To solve this, an attempt was made to employ electrostatic doping to inject free carriers, which form free trions, and successfully observe the trion funneling effects in suspended MoSe_2_ monolayer [26]. However, these results have only been observed at cryogenic temperatures. Therefore, to emphasize the practical aspects of trion transport applications, it is essential to demonstrate trion motion under the influence of an electric field at room temperature.

Here, we achieved trion drift in a suspended WS_2_ monolayer via a gate-tunable electric field at room temperature. Since the WS_2_ monolayer is connected to the top contact, applying a finite bottom-gate voltage leads to electrostatic doping in the WS_2_ monolayer. This electrostatic doping introduces additional charge carriers into the system, enabling the formation of trions. Additionally, applying a gate voltage creates an electric field in the vicinity of the suspended monolayer. Under the influence of the electric field, trions funnel toward the center of the suspended monolayer, and this was analyzed by comparing trion emission under local excitation with different excitation spots. The drift of locally excited trions under the influence of the electric field was reproducible through finite element method (FEM) simulations, assuming the same conditions as the experiment, further validating trion drift effect.

## 2 Results

### 2.1 Device characterization

**Figure 1:**
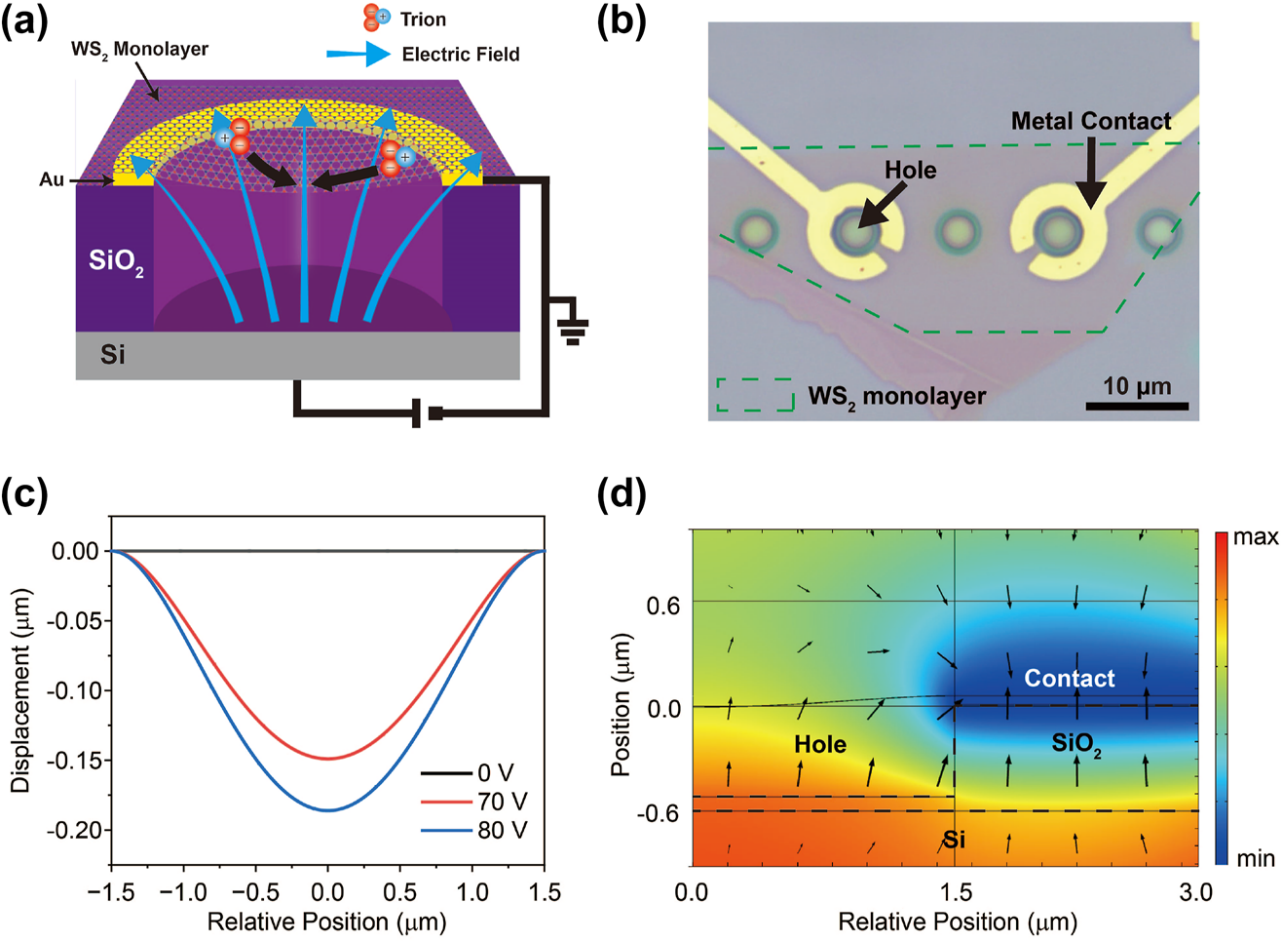
Operating principle of the suspended WS_2_ monolayer device. (a) Schematic illustration of trion funneling under the influence of electric force and electrostatic gating. (b) Optical microscope image of the fabricated sample. The area marked by a dashed line represents the WS_2_ monolayer. The scale bar indicates 10 μm. (c) Simulated displacement of the WS_2_ monolayer at different bottom-gate voltages, showing an increase with higher voltage. (d) Cross-sectional view of the simulated electric potential distribution (shown in colors) and electric field distribution (shown in arrows).

Figure 1a depicts the schematic illustration of our device structure and operating mechanism. Our device consists of a WS_2_ monolayer suspended over a hole-shaped trench with a metallic contact (see Figure 1b) consisting of Ti (8 nm) and Au (30 nm). When a voltage was applied to the bottom gate, while maintaining the top contact at ground potential, two effects were induced. First, since the monolayer is connected to the top contact, it undergoes electrostatic doping, similar to the operation of a conventional field-effect transistor (FET) [21]. Upon positive gate-voltage, electrons are injected, leading to the formation of negatively charged trions. Second, due to the difference in electric potential across the vertical direction (see Figure 1c), an electric field is induced around the WS_2_ monolayer, which causes the trions to drift in the opposite direction. Moreover, circular shape metallic contact enables the electric field exhibits radial symmetry, which causes the trions to drift toward the center of the suspended monolayer. Taken together, as a result of these two effects, we were able to convert neutral excitons into trions and control their motion simultaneously. Additionally, the suspended monolayer as a whole can also experience electrostatic forces, which cause it to deform in the downward direction [31], [32]. Due to the top and bottom geometry of our device, an electric field is formed that primarily exhibits vertical directionality. We believe that this deformation causes the monolayer’s orientation to align more with the vertical direction of the electric field, thereby increasing the intensity of the effective electric force experienced by the trions in the monolayer.

To quantitatively understand the operation of the device, we simulated the behavior of a suspended WS_2_ monolayer at various bottom-gate voltages using the FEM method. For these simulations, we used the mechanical properties of the monolayer, including its Young’s modulus, Poisson’s ratio, and thickness, among others [33]. For simplicity, we assumed that the suspended monolayer remained flat at 0 V and utilized the system’s rotational symmetry for computational convenience. Figure 1c shows the calculated distribution of electric potential and the corresponding field distribution at 80 V, presented in a cross-sectional view. As previously noted, the electric field spreads radially from the center of the suspended region. Figure 1d shows the calculation results of monolayer displacement at different bottom-gate voltages, demonstrating that the monolayer is pulled downward as the voltage increases.

### 2.2 Gate-voltage dependence of the response in suspended WS_2_ monolayer

**Figure 2:**
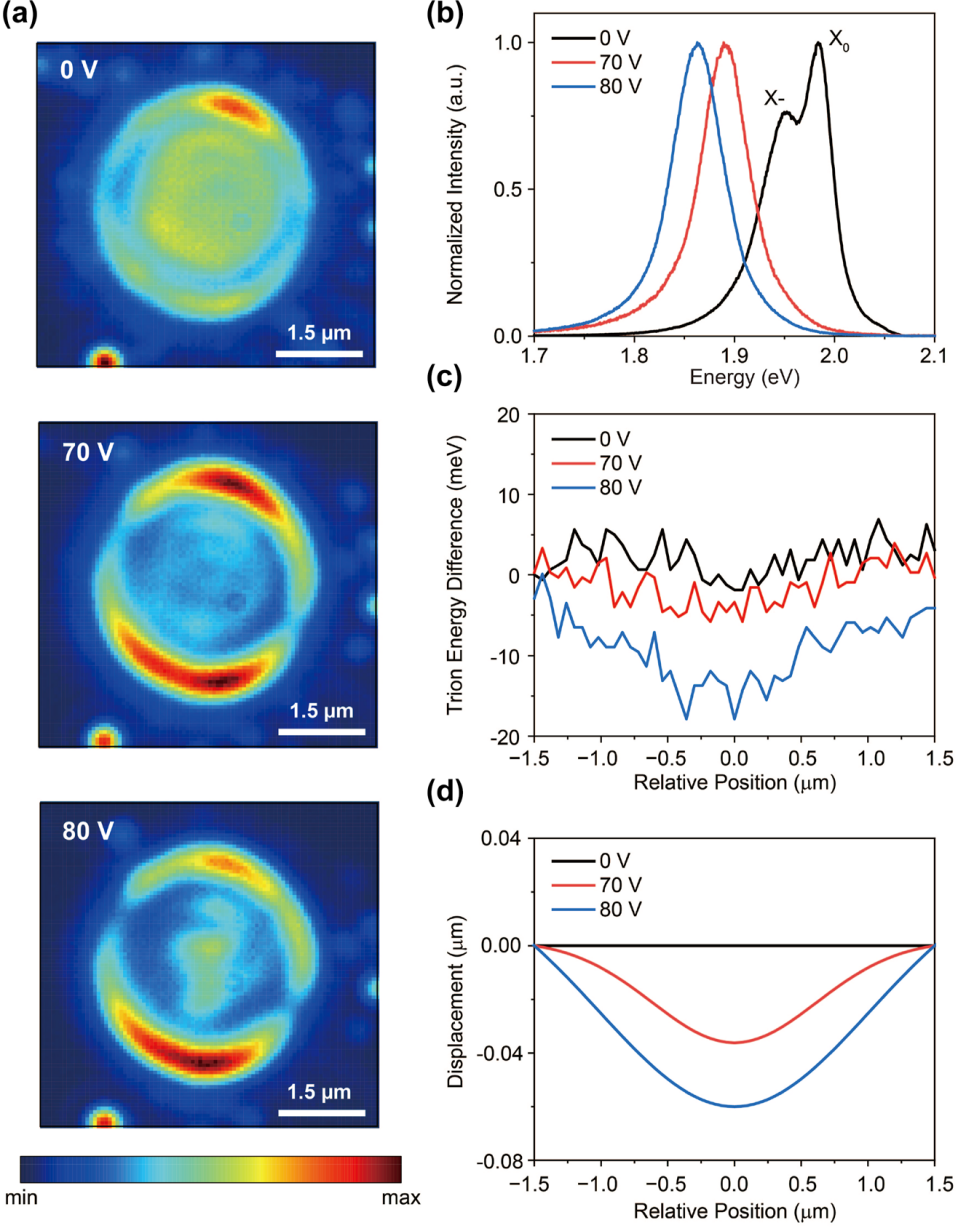
The suspended WS_2_ monolayer’s response to increasing gate voltage. (a) Changes in the spatial distribution of trion PL at three different voltages. As the voltage increases, the trion PL intensity becomes more concentrated at the center of the suspended layer. The scale bar indicates 1.5 μm. (b) Changes in the PL spectrum at three different voltages, measured at the hole center. At 0 V, there are two peaks for exciton and trion, but above 70 V, all excitons are converted to trions, leaving only a single emission peak. (c) Spatial distribution of trion energy difference relative to the flat surface in the suspended layer at various gate voltages. As the voltage increases, the strain at the center of the layer also increases, causing the trion energy to be lowest at the center. (d) Displacement of the monolayer WS_2_ at 0 V, 70 V and 80 V estimated from the trion energy distribution. The displacement is calculated through the strain formula, which corresponds to the geometry of our experiment conditions [34], [35], [36].

To experimentally validate these results, we employed a continuous-wave (CW) laser with a wavelength of 594 nm to monitor the changes in monolayer photoluminescence (PL) as the gate voltage was increased. Figure 2a shows the spatial distribution of trion PL at the suspended monolayer for bottom-gate voltages of 0 V, 70 V, and 80 V, respectively. The bright PL at the edge of the etched hole is attributed to the plasmonic enhancement of trion PL and substantial strain in that region. These images were obtained by exciting the entire monolayer region with an enlarged laser spot. In addition, Figure 2b shows the PL spectrum at each voltage. At 0 V, both the exciton and negative trion peaks were observed, which can be attributed to the initial *n*-doping of WS_2_. Upon increasing the gate voltage to 70 V or higher, only a single peak was observed, indicating the complete conversion of neutral excitons to trions due to electrostatic doping. (See Figure 7 in Appendix C for a detailed PL development over increasing voltage). By comparing 0 V and 80 V results in Figure 2a, we observe that at 0 V, the trion PL intensity is relatively uniform (To distinguish the trion PL from exciton at 0 V, an optical filter was used), whereas at 80 V, the intensity increases at the center of the hole. This implies that the trions are drifting toward the hole center at 80 V.

For further analysis, we also acquired the PL spectral distribution to obtain the trion energy difference in the suspended area (see Figure 2c). At 0 V, the trion energy distribution exhibited a slight red shift, suggesting that the monolayer is nearly flat with minimal initial strain. However, as the voltage increased to 70 V or higher, the energy at the center of the hole decreased radially, displaying a clear red shift. Given that tensile strain reduces the optical band gap in TMD materials [34], [35], this suggests that as the voltage increases, the monolayer is pulled downward, which is in good agreement with the simulation results. We also estimated the displacement of the WS_2_ monolayer at 80 V using the strain expression *ε* = 2/3(*h*/*a*)^2^ [36] corresponding to the geometry of our experimental environment. (*a* is the radius of a hole and *h* is the maximum displacement.)

### 2.3 Funneling of locally excited trion in suspended WS_2_ monolayer

**Figure 3:**
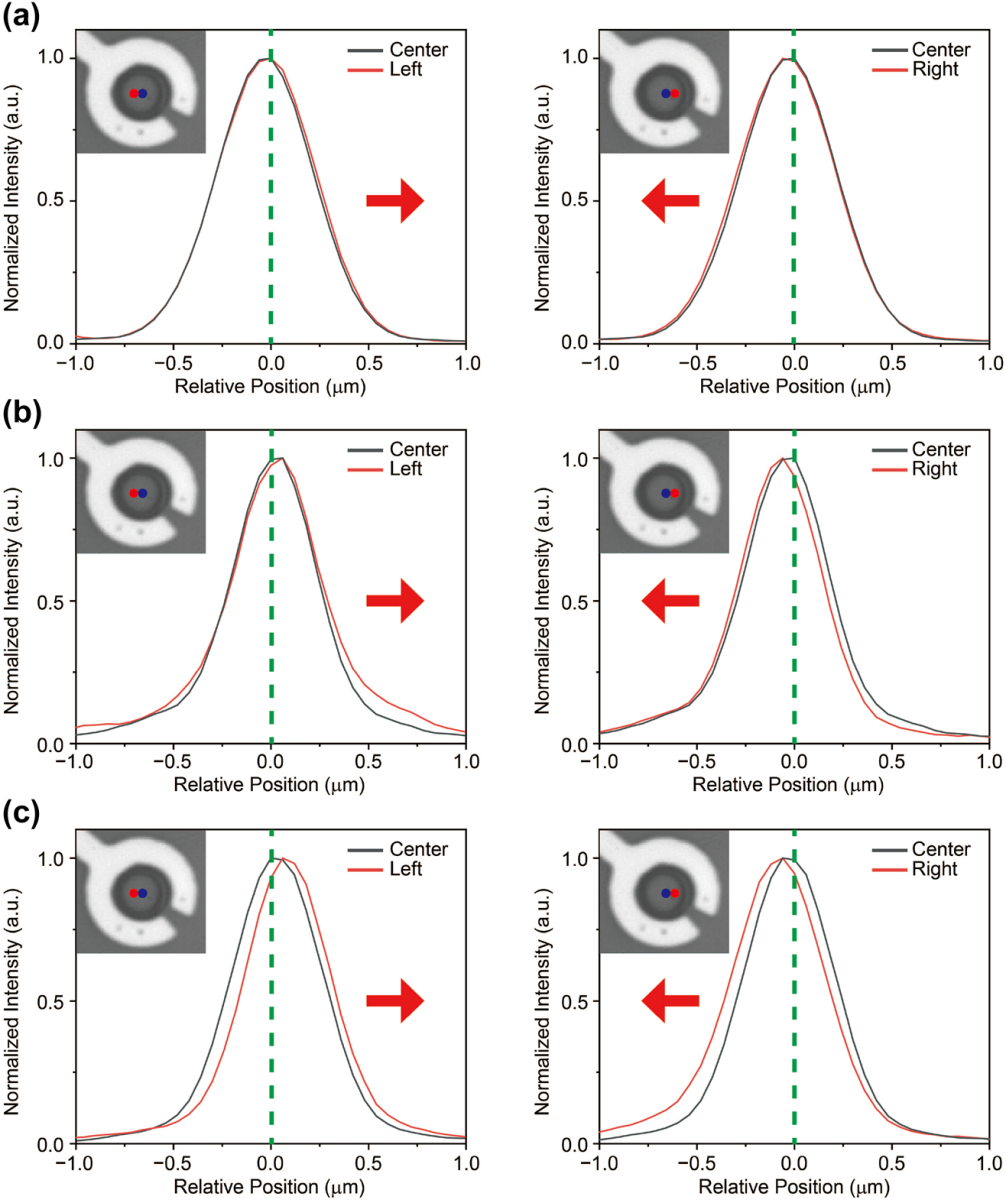
Observation of the drift of trions toward the center of the hole. (a–c) Comparison of trion PL distribution along the horizontal direction in the suspended monolayer at gate voltages of 0 V, 70 V and 80 V. Insets show CCD images of the sample, with blue and red circles indicating two different excitation locations separated by approximately 600 nm. Each figure shows two different PL distributions: one excited at the center and the other at positions displaced in the left and right directions, allowing for a comparison of the PL behavior along the horizontal axis at increasing gate voltages. The gray solid line represents the PL at the center, while the red solid line corresponds to the PL at the left and right positions. There is no significant change in the PL distribution at 0 V; however, as the voltage increases, the PL distribution shifts toward the center of the hole (the red arrow indicates the center direction).

The response of the monolayer to increasing voltage, including both exciton-to-trion conversion and intensity increase at the hole center, suggests that trions accumulate at the center of the hole. To clarify the trion’s motion, we locally excited trions at various locations and examined the changes in spatial PL distribution for different excitation positions as the gate voltage increased. We compared the spatial distribution of trion PL excited at the hole center and at locations 600 nm to the left and right at each voltage. At 0 V, no significant change is observed in the trion PL distribution (see Figure 3a). In both cases of left and right excitations, the trion PL exhibits a symmetric Gaussian distribution. This observation suggests that, in the absence of external influences such as applied forces, trions will naturally diffuse in all directions driven by concentration gradients [6], [37]. However, at 70 V, the trion PL distribution on both sides appears to be slightly drift toward the hole center (see Figure 3b). The PL distribution at the hole center maintains a Gaussian profile, as observed at 0 V, whereas the PL distribution on left and right excitation is skewed toward the hole center. Moreover, this trend becomes more pronounced at 80 V (see Figure 3c). Based on the observed asymmetric PL distribution toward the hole center with increasing gate voltage, along with the augmentation of this effect at higher voltages, provides evidence of trion drift induced by the electric field. Here, we present only the results in the horizontal direction; however, similar trends are observed in the vertical direction as well (see Figure 5 in Appendix A), and both results demonstrate trion funneling toward the center in the radial direction.

### 2.4 Theoretical modeling of trion drift using the finite element method

To validate the experimental results of trion drift, we conducted numerical simulations using 1-dimensional drift-diffusion equations to determine whether they lead to similar conclusions. The equation is expressed as follows:

$$D\frac{\partial^{2}n_{T}}{\partial x^{2}}+\mu \frac{\partial }{\partial x}\left(n_{T}\left(\frac{\partial \phi }{\partial x}+eE\right)\right)+I\left(x\right)-\frac{n_{T}}{\tau }=\frac{\partial n_{T}}{\partial t}$$

The notation used in the equation is explained as follows: *D* is the diffusion coefficient, *n*_*T*_ is the local concentration, *μ* is the mobility, *τ* is the lifetime, *ϕ* is the potential energy, *E* is the electric field, and *I* is the source profile of the trions. The first term on the left-hand side of the equation represents the diffusion of the trions, while the second term describes their drift motion. In our experimental setup, the external forces that could cause the drift motion in the second term are the potential energy gradient and the electric force, denoted by ∂*ϕ*/∂*x* and *eE*, respectively. The potential energy was directly obtained from the trion energy distribution measured in previous experiments. To calculate the electric force, we estimated the potential difference between the top and bottom gates by analyzing the experimentally obtained strain distribution of the monolayer and utilized the corresponding in-plane electric field distribution. The source profile was derived from the pumping laser used in the experiment, which exhibits a Gaussian distribution. Since a CW laser was used to excite the trions, the right-hand side of the equation is set to zero when solving it. Finally, we set the values of *D* to be 0.2 cm^2^/s and *τ* to be 10 ps to reproduce the experimental data. We emphasize that if excitons remain in the system, their motion must be considered, as they can diffuse and recombine with electrons to form trions. In such cases, coupled equations describing both excitons and trions must be solved [24]. However, in our case, all excitons are fully converted into trions at gate voltages of 70 V or higher. Thus, it is sufficient to solve a single equation to analyze trion’s motion.

**Figure 4:**
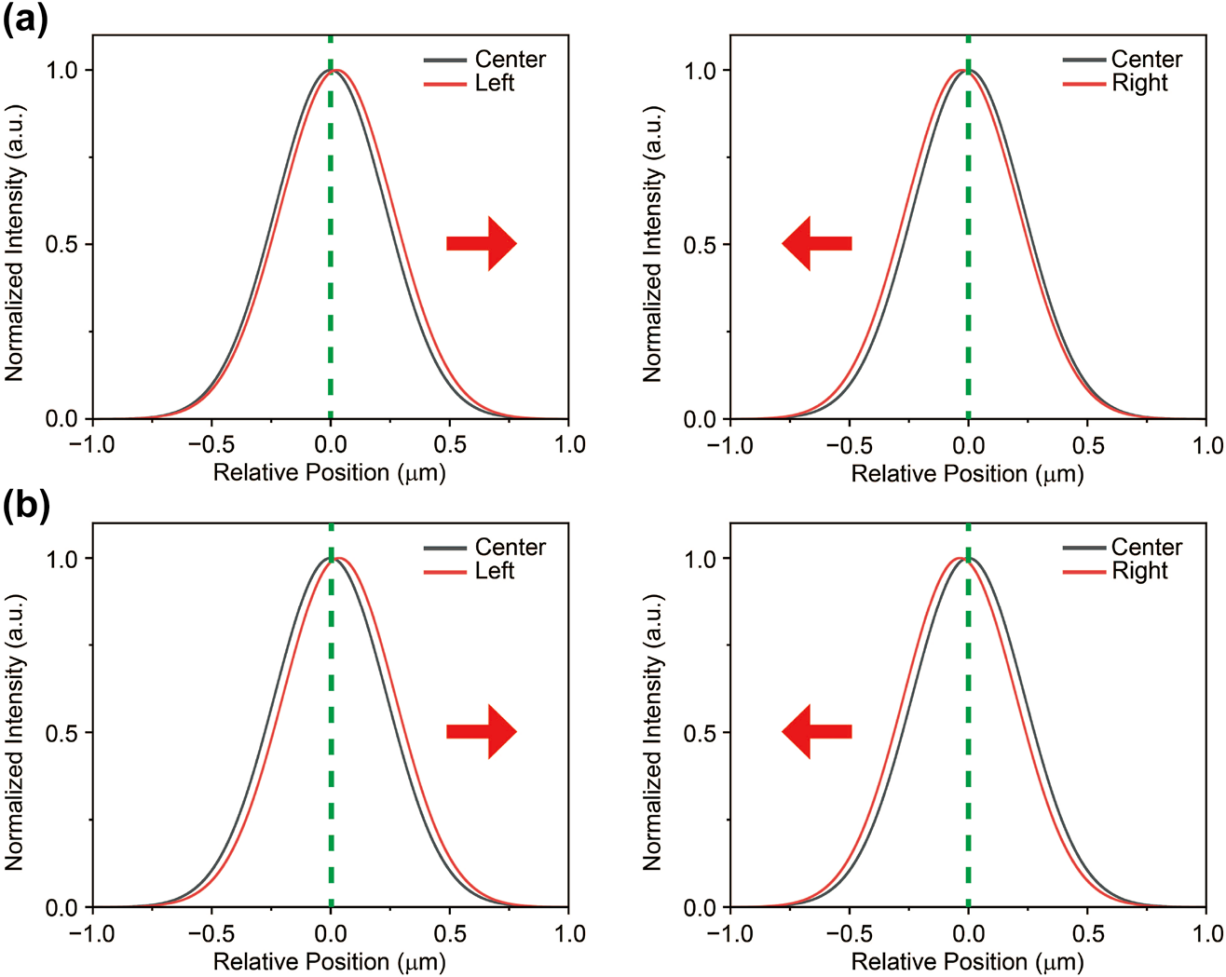
Numerically calculated spatial distribution of trion population using drift-diffusion equations. (a–b) A comparison of trion concentration along the horizontal direction at different gate voltages, which corresponds to the experimental results. Black and red solid lines represent the trion concentration at the center and at the deviated position (the red arrow indicates the center direction). The simulated results qualitatively reproduce the experimental data, as shown by the trion concentration calculated with the drift-diffusion model in the presence of an in-plane electric field.

**Figure 5:**
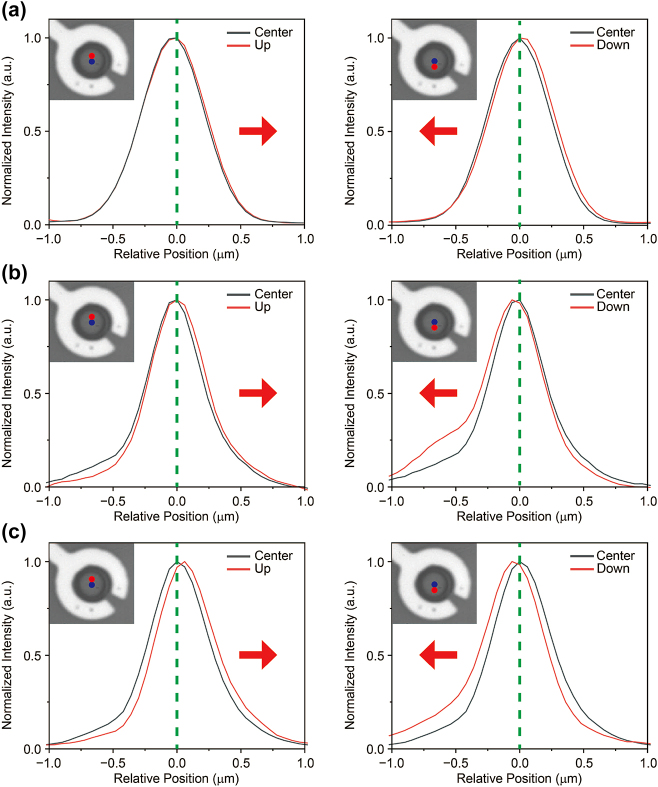
Observation of the drift of trions toward the center of the hole. (a–c) Comparison of trion PL distribution along the vertical direction in the suspended monolayer at gate voltages of 0 V, 70 V and 80 V. Insets show CCD images of the sample, with blue and red circles indicating two different excitation locations separated by approximately 600 nm. Each figure shows two different PL distributions: one excited at the center and the other at positions displaced in the up and down directions, allowing for a comparison of the PL behavior along the vertical axis at increasing gate voltages. The gray solid line represents the PL at the center, while the red solid line corresponds to the PL at the up and down positions. There is no significant change in the PL distribution at 0 V; however, as the voltage increases, the PL distribution shifts toward the center of the hole (the red arrow indicates the center direction).

**Figure 6:**
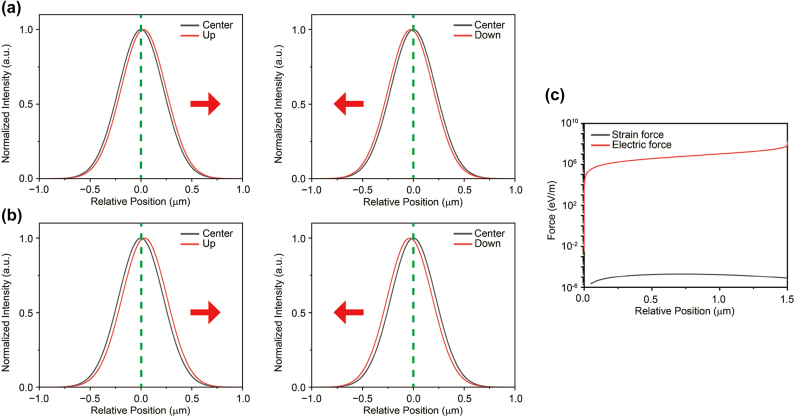
Numerically calculated spatial distribution of trion population using drift-diffusion equations. (a–b) A comparison of trion concentration along the vertical direction at different gate voltages, which corresponds to the experimental results. Black and red solid lines represent the trion concentration at the center and at the deviated position (the red arrow indicates the center direction). The simulated results qualitatively reproduce the experimental data, which is consistent with the main data in Figure 4. (c) Comparison between strain-induced force (Black) and electric force (Red) as a function of radial distance from the center at 80 V (the forces are directed toward the center). Since the displacement of the layer is marginal even at 80 V, the effective force due to strain is negligible in comparison to the electric force.

**Figure 7:**
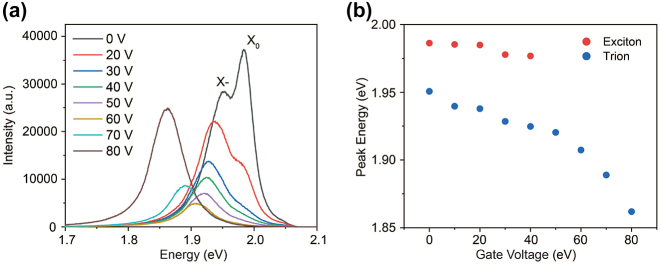
In-depth variations of PL spectrum with respect to gate-voltage. (a) Changes in the PL spectrum at various voltages. At 0 V, there are two peaks for excitons (*X*_0_) and trions (*X*−). As the voltage increases, the exciton peak is gradually diminished and vanishes at around 50 V, leaving only the trion peak. (b) Gate-voltage dependence of exciton and trion energies. Typically, exciton exhibits a blue shift with increasing gate voltage [41]. However, we have observed red shift behavior, which may come from strain effect.

**Figure 8:**
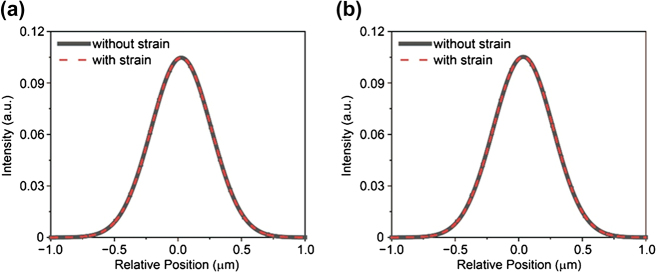
Numerical simulation results of trion concentration with and without the influence of strain using drift-diffusion equations. (a–b) Comparison of results regarding the presence or absence of strain-induced force, specifically for left excitation, at 70 V and 80 V, respectively. The potential gradient was directly fitted using the experimental spectrum presented in Figure 2c. For both gate voltages, the simulation results show no significant change under the influence of strain.

Figure 4a shows the simulated results of local concentrations at 70 V, assuming the same excitation position as in the experiment. To simulate different excitation positions, we shifted the source profile in the equation by 600 nm to the left and right relative to the center. The simulation results at 70 V show a shift of the trion profile toward the center, similar to the experimental observations. At 80 V, the results were also consistent with the experimental findings (see Figure 4b), and the simulation confirmed that the drift effect increases with the voltage. In addition to the horizontal directions (left and right), similar calculations were conducted in the vertical direction (up and down), yielding consistent trends (see Figure 6 in Appendix B). These findings indicate that the trion behaves like a free particle, capable of moving under the influence of an electric field. However, one additional aspect that needs to be verified is the influence of the energy gradient on the second term of the equation. Since it is not possible to separate the strain effect from the electric field in our system, it is crucial to identify the dominant factor driving the trion drift, as the strain can also generate an effective force that could contribute to the trion’s movement. For this reason, we compared the two forces and found that the strain-induced force is negligible compared to the electric force (see Figure 6 in Appendix B). Additionally, we were able to separate the two contributions (strain-induced potential energy gradient and electric force) in the drift-diffusion equation to assess the extent to which strain influences the simulation outcomes. For both the 70 V and 80 V, the results of this comparison indicated that strain had no significant effect. (See Figure 8 in Appendix D). Consequently, we conclude that the contribution of the strain-induced force is marginal, confirming that the electric force is the dominant factor in trion motion.

## 3 Conclusions

Here, we demonstrate the observation of trion drift at room temperature in suspended monolayer WS_2_ under increasing gate voltages. Our system enables complete conversion of excitons into trions under non-resonant excitation while simultaneously applying an electric field. With an increasing gate voltage, we observed a gradual increase in trion drift by analyzing the PL distribution and further validated the result using a diffusion model with an electric field. We emphasized that room temperature operation is possible for several reasons. Based on previous experiments in low-temperature environments [23], [24], [25], [26], it is expected that fully converting excitons into trions through doping in a room-temperature environment could enable the observation of trion drift. However, in WSe_2_ and MoSe_2_, trions were not detected at room temperature due to thermal fluctuations [21], [38]. In MoS_2_, while trions could be generated via electrostatic doping, complete conversion of excitons into trions was not achieved [39]. Consequently, we focused on WS_2_, where excitons are fully converted into trions at high gate voltages [22]. Additionally, the suspended geometry may contribute to the successful observation of trion drift at room temperature. By reducing surface roughness and minimizing scattering processes from the substrates, the suspended geometry could enhance the lifetime of trions [40]. This effect is particularly significant despite trion-phonon scatterings that inhibit trion transport at higher temperatures [21], [38]. We believe that our findings could potentially be improved by using high-purity TMD materials, as it has been reported that reducing defects increases the trion lifetime [37]. It is worth noting that, in contrast to previous studies that observed trion drift primarily at cryogenic temperatures, our findings show a pronounced trion drift at room temperature, which provides an intriguing platform for exploring the trion physics and their potential applications in trion-based optoelectronic devices.

## 4 Methods

### 4.1 Sample fabrication

The circular array pattern with a diameter of 3 μm was fabricated on a silicon/silicon oxide (600 nm) substrate coated with positive photoresist using electron beam lithography and then etched to a depth of 520 nm using a wet etching process with buffered oxide etchant (BOE). The metallic contact pattern surrounding the holes was fabricated using electron beam lithography, followed by the deposition of gold (Au) and titanium (Ti) with thicknesses of 30 nm and 8 nm, respectively, through thermal evaporation. A monolayer of WS_2_ was mechanically exfoliated from bulk crystals (2D Semiconductors) and subsequently transferred onto a patterned substrate via a dry transfer technique. The substrate was attached to a printed circuit board (PCB) using an electrically conductive adhesive, and the substrate and PCB were connected via wire bonding.

### 4.2 Experimental measurement

The fabricated sample was placed inside a chamber containing an objective lens with 100× magnification and a numerical aperture (NA) of 0.9, where the temperature was maintained at room temperature (295 K) and the internal pressure was kept at a medium vacuum of 0.6 Torr. All experiments were conducted under these temperature and pressure conditions. To measure photoluminescence (PL), a continuous-wave (CW) laser with a wavelength of 594 nm was focused onto the WS_2_ monolayer to excite the excitons and trions, with the laser pumping power fixed at 50 μW. To apply a bottom-gate voltage, the PCB was subsequently connected to a source-measurement unit (SMU, Keithley 2612B). The trion PL emission, collected through a lens, was sent to a spectrometer (Princeton Instruments, HRS-300) and a CCD camera (PIXIS 400) for spectrum and image analysis. To investigate the spatial distribution of trion PL image, a lens was inserted to increase the pumping spot size for uniform excitation across the entire suspended monolayer.

### 4.3 FEM simulation

The electromechanical response of the suspended WS_2_ monolayer as a function of gate voltage was computed using the electrostatics and solid mechanics interfaces within COMSOL Multiphysics software, which employs the finite element method (FEM) for simulation. The mechanical properties of the WS_2_ monolayer were set with a density of 7.5 g/cm^3^, Young’s modulus of 302.4 GPa, and Poisson’s ratio of 0.22 [33]. Additionally, the electric field applied in the in-plane direction to the monolayer was obtained using the Electrostatics interface, while the one-dimensional drift-diffusion equation was solved employing the Classical PDE interface.

## Appendix B: Simulated results in vertical direction and effective force comparison

See Figure 6.

## Appendix C: Detailed changes of PL spectrum with increasing gate-voltage

See Figure 7.

## Appendix D: Comparison of simulation results with and without strain effects

See Figure 8.

## References

[1] Mak K. F., Lee C., Hone J., Shan J., Heinz T. F. (2010). Atomically thin MoS_2_: a new direct-gap semiconductor. *Phys. Rev. Lett.*.

[2] Radisavljevic B., Radenovic A., Brivio J., Giacometti V., Kis A. (2011). Single-layer MoS_2_ transistors. *Nat. Nanotechnol*..

[3] Wang Q. H., Kalantar-Zadeh K., Kis A., Coleman J. N., Strano M. S. (2012). Electronics and optoelectronics of two-dimensional transition metal dichalcogenides. *Nat. Nanotechnol*..

[4] Splendiani A. (2010). Emerging photoluminescence in monolayer MoS_2_. *Nano Lett*..

[5] Arora A., Nogajewski K., Molas M., Koperski M., Potemski M. (2015). Exciton band structure in layered MoSe_2_: from a monolayer to the bulk limit. *Nanoscale*.

[6] Uddin S. Z. (2020). Neutral exciton diffusion in monolayer MoS_2_. *ACS Nano*.

[7] Lee J. (2021). Switchable, tunable, and directable exciton funneling in periodically wrinkled WS_2_. *Nano Lett*..

[8] San-Jose P., Parente V., Guinea F., Roldán R., Prada E. (2016). Inverse funnel effect of excitons in strained black phosphorus. *Phys. Rev. X*.

[9] Moon H. (2020). Dynamic exciton funneling by local strain control in a monolayer semiconductor. *Nano Lett*..

[10] High A. A., Novitskaya E. E., Butov L. V., Hanson M., Gossard A. C. (2008). Control of exciton fluxes in an excitonic integrated circuit. *Science*.

[11] Grosso G. (2009). Excitonic switches operating at around 100 K. *Nat. Photonics*.

[12] Dirnberger F. (2021). Quasi-1D exciton channels in strain-engineered 2D materials. *Sci. Adv.*.

[13] Li H. (2015). Optoelectronic crystal of artificial atoms in strain-textured molybdenum disulphide. *Nat. Commun*..

[14] Harats M. G., Kirchhof J. N., Qiao M., Greben K., Bolotin K. I. (2020). Dynamics and efficient conversion of excitons to trions in non-uniformly strained monolayer WS_2_. *Nat. Photonics*.

[15] Liu Y., Dini K., Tan Q., Liew T., Novoselov K. S., Gao W. (2020). Electrically controllable router of interlayer excitons. *Sci. Adv.*.

[16] Unuchek D., Ciarrocchi A., Avsar A., Watanabe K., Taniguchi T., Kis A. (2018). Room-temperature electrical control of exciton flux in a van der Waals heterostructure. *Nature*.

[17] Sun Z. (2022). Excitonic transport driven by repulsive dipolar interaction in a van der Waals heterostructure. *Nat. Photonics*.

[18] Rivera P. (2016). Valley-polarized exciton dynamics in a 2D semiconductor heterostructure. *Science*.

[19] Fowler-Gerace L. H., Zhou Z., Szwed E. A., Choksy D. J., Butov L. V. (2024). Transport and localization of indirect excitons in a van der Waals heterostructure. *Nat. Photonics*.

[20] Yuan L. (2020). Twist-angle-dependent interlayer exciton diffusion in WS_2_–WSe_2_ heterobilayers. *Nat. Mater*..

[21] Ross J. S. (2013). Electrical control of neutral and charged excitons in a monolayer semiconductor. *Nat. Commun*..

[22] Shang J. (2015). Observation of excitonic fine structure in a 2D transition-metal dichalcogenide semiconductor. *ACS Nano*.

[23] Sanvitto D. (2001). Observation of charge transport by negatively charged excitons. *Science*.

[24] Pulizzi F. (2003). Optical imaging of trion diffusion and drift in GaAs quantum wells. *Phys. Rev. B*.

[25] Cheng G., Li B., Jin Z., Zhang M., Wang J. (2021). Observation of diffusion and drift of the negative trions in monolayer WS_2_. *Nano Lett*..

[26] Lee S. W. (2023). Electric-field-driven trion drift and funneling in MoSe_2_ monolayer. *Nano Lett*..

[27] Zhang Q., Sun H., Tang J., Dai X., Wang Z., Ning C. Z. (2022). Prolonging valley polarization lifetime through gate-controlled exciton-to-trion conversion in monolayer molybdenum ditelluride. *Nat. Commun*..

[28] Lee H. (2023). All-optical control of high-purity trions in nanoscale waveguide. *Nat. Commun*..

[29] Lee H. (2022). Drift-dominant exciton funneling and trion conversion in 2D semiconductors on the nanogap. *Sci. Adv.*.

[30] Kang M. (2024). Nanoscale manipulation of exciton-trion interconversion in a MoSe_2_ monolayer via tip-enhanced cavity-spectroscopy. *Nano Lett*..

[31] Prasad P., Arora N., Naik A. K. (2017). Parametric amplification in MoS_2_ drum resonator. *Nanoscale*.

[32] Kumar A. M. (2024). Strain fingerprinting of exciton valley character in 2D semiconductors. *Nat. Commun*..

[33] Falin A. (2021). Mechanical properties of atomically thin tungsten dichalcogenides: WS_2_, WSe_2_, and WTe_2_. *ACS Nano*.

[34] Niehues I. (2018). Strain control of exciton–phonon coupling in atomically thin semiconductors. *Nano Lett*..

[35] Lloyd D. (2016). Band gap engineering with ultralarge biaxial strains in suspended monolayer MoS_2_. *Nano Lett*..

[36] Small M. K., Nix W. D. (1992). Analysis of the accuracy of the bulge test in determining the mechanical properties of thin films. *J. Mater. Res*..

[37] Kim B. (2022). Free trions with near-unity quantum yield in monolayer MoSe_2_. *ACS Nano*.

[38] Liu M. (2023). Periodical ripening for MOCVD growth of large 2D transition metal dichalcogenide domains. *Adv. Funct. Mater*..

[39] Mak K. F. (2013). Tightly bound trions in monolayer MoS_2_. *Nat. Mater*..

[40] Sharma A. (2022). Engineering the dynamics and transport of excitons, trions, and biexcitons in monolayer WS_2_. *ACS Appl. Mater. Interfaces*.

[41] Zhu B., Chen X., Cui X. (2015). Exciton binding energy of monolayer WS_2_. *Sci. Rep*..

